# Supplementary material for: A plant cell death-inducing protein from litchi interacts with Peronophythora litchii pectate lyase and enhances plant resistance
Source: Nat Commun. 2024 Jan 2;15:22. doi: 10.1038/s41467-023-44356-y (PMC10761943; doi:10.1038/s41467-023-44356-y)
Supplement: Supplementary file 1 — Supplementary Information [file 41467_2023_44356_MOESM1_ESM.pdf]

Supplementary Information for

A plant cell death-inducing protein from litchi interacts with *Peronophythora litchii* pectate lyase and enhances plant resistance

Wen Li[1, #], Peng Li[1, #], Yizhen Deng[2], Junjian Situ[1], Zhuoyuan He[3], Wenzhe Zhou[1], Minhui Li[1], Pinggen Xi[1], Xiangxiu Liang[3], Guanghui Kong[1*], Zide Jiang[1*]

[*] Correspondence:

Zide Jiang (zdjiang@scau.edu.cn)

Guanghui Kong (gkong@scau.edu.cn)

[#] These authors contributed equally.

This PDF file includes: 15 Supplementary Figures.

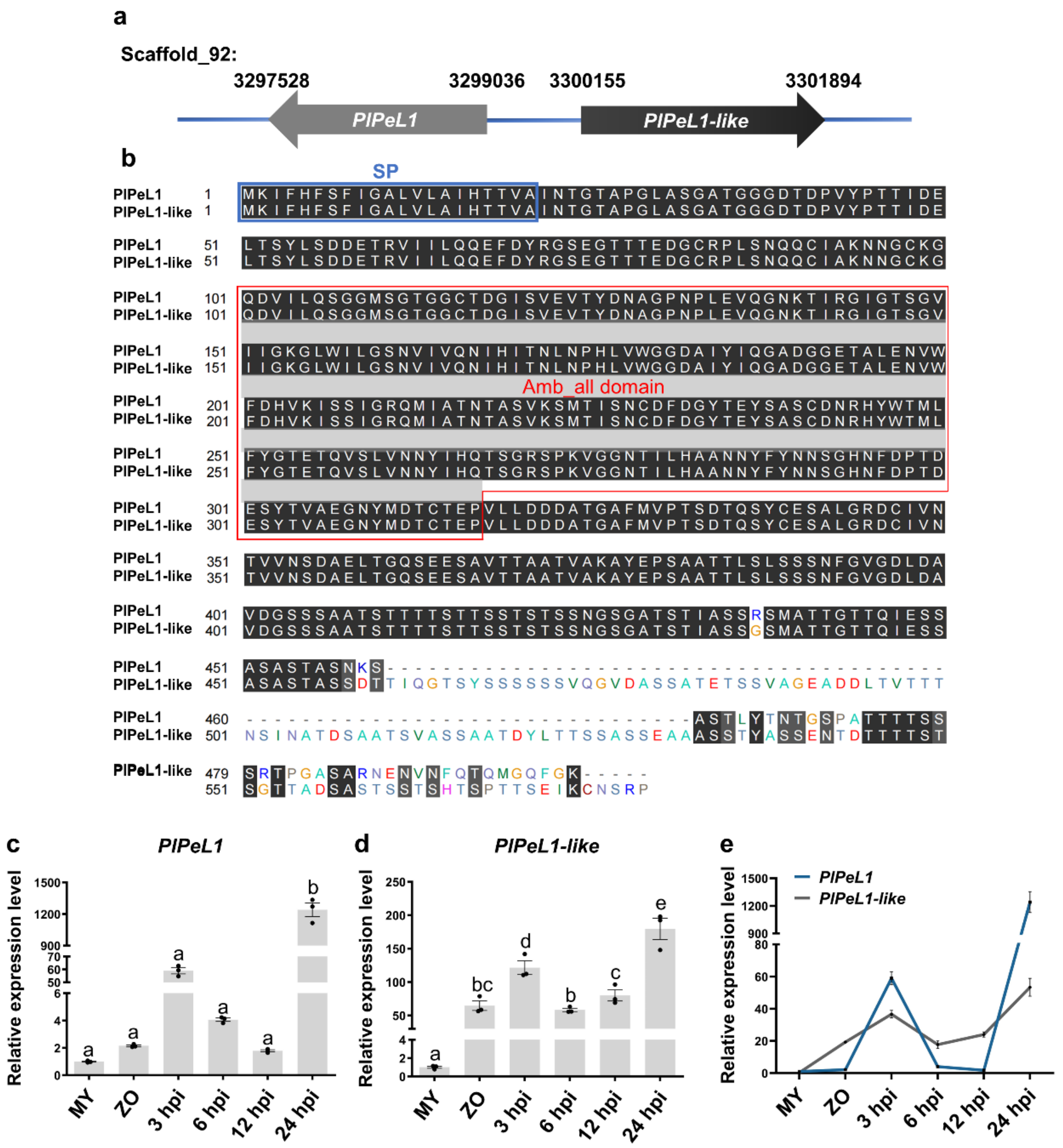

Supplementary Fig. 1. *PlPeL1* and *PlPeL1-like* arrange in a head-to-head reversed complement fashion in genome and their expression pattern analysis.

(a) PlPeL1 and PlPeL1-like located on the scaffold_92: 3297528-3299036(-) and scaffold_92: 3300155-3301894(+), respectively. (b) Protein sequence alignment of PlPeL1 and PlPeL1-like. Black and gray indicate identical and similar amino acid sequences, respectively. The blue box indicates the signal peptide, and the red box indicates the Amb_all domain. (c,d) Expression pattern of *PlPeL1* and *PlPeL1-like* genes. Expression levels were determined by quantitative reverse-transcription PCR

(qRT-PCR) using cDNA of vegetative mycelia (MY), zoospores (ZO), and samples from 3, 6, 12, and 24 h post-inoculation (hpi) with zoospores on leaves. The relative expression level of MY was set as 1. *PlActin* was used as the endogenous control. Data are shown as the mean ± SE of three replicates. The data were statistically analyzed with SPSS (version 20.0) with one-way ANOVA and means followed by different letters are significantly different ($P < 0.05$). (e) Conduct a comparative analysis of the expression levels of *PlPeL1* and *PlPeL1-like*. Calculate the relative expression levels of *PlPeL1* and *PlPeL1-like* at different stages, with the expression level of *PlPeL1* at the MY stage set as the reference (normalized to 1). *PlActin* was used as the endogenous control. Data are shown as the mean ± SE of three replicates. These experiments were repeated three times with similar results. Source data are provided as a Source Data file.

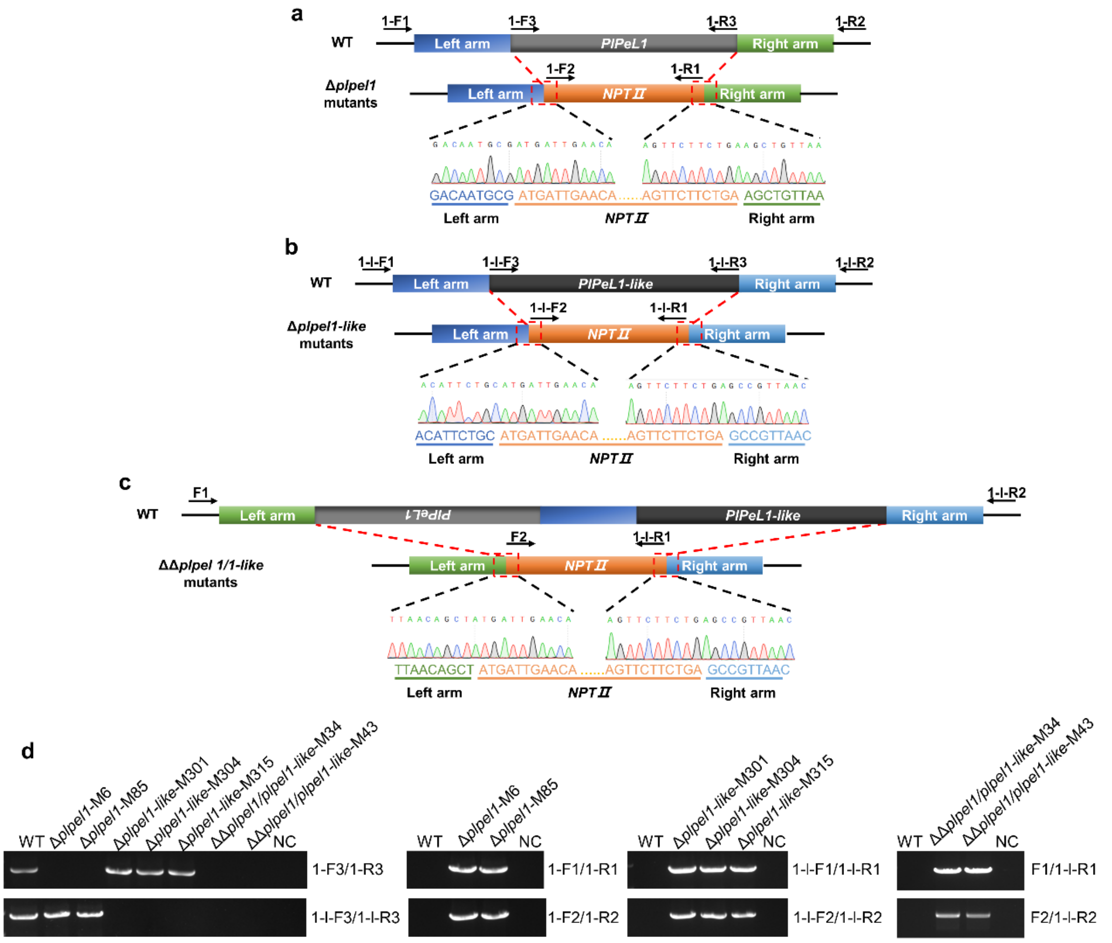

Supplementary Fig. 2. Schematic diagram showing CRISPR/Cas9-mediated knockout of *PlPeL1* and *PlPeL1-like*.

(a-c) The schematic diagram showed the strategy generating Δ*plpel1*, Δ*plpel1-like*, and ΔΔ*plpel1/plpel1-like* mutants by CRISPR/Cas9 technique, in which the *NPTII* gene replaces the targets. The primers used for PCR analysis are indicated by horizontal arrows, and were listed in Supplementary Data 4. (d) Genomic DNA PCR analysis of the Δ*plpel1*, Δ*plpel1-like*, and ΔΔ*plpel1/plpel1-like* mutants. On the right side of each image are the primer pairs used and primers were shown in (a-c). Δ*plpel1*-M6 and Δ*plpel1*-M85 were knockout mutants of *PlPeL1* gene. Δ*plpel1-like*-M301, Δ*plpel1-like*-M304, and Δ*plpel1-like*-M315 were knockout mutants of *PlPeL1-like* gene. ΔΔ*plpel1/plpel1-like*-M34 and ΔΔ*plpel1/plpel1-like*-M43 were knockout mutants of

PlPeL1 and *PlPeL1-like* genes. Negative control (NC), ddH_2O was used as template in the PCR reaction.

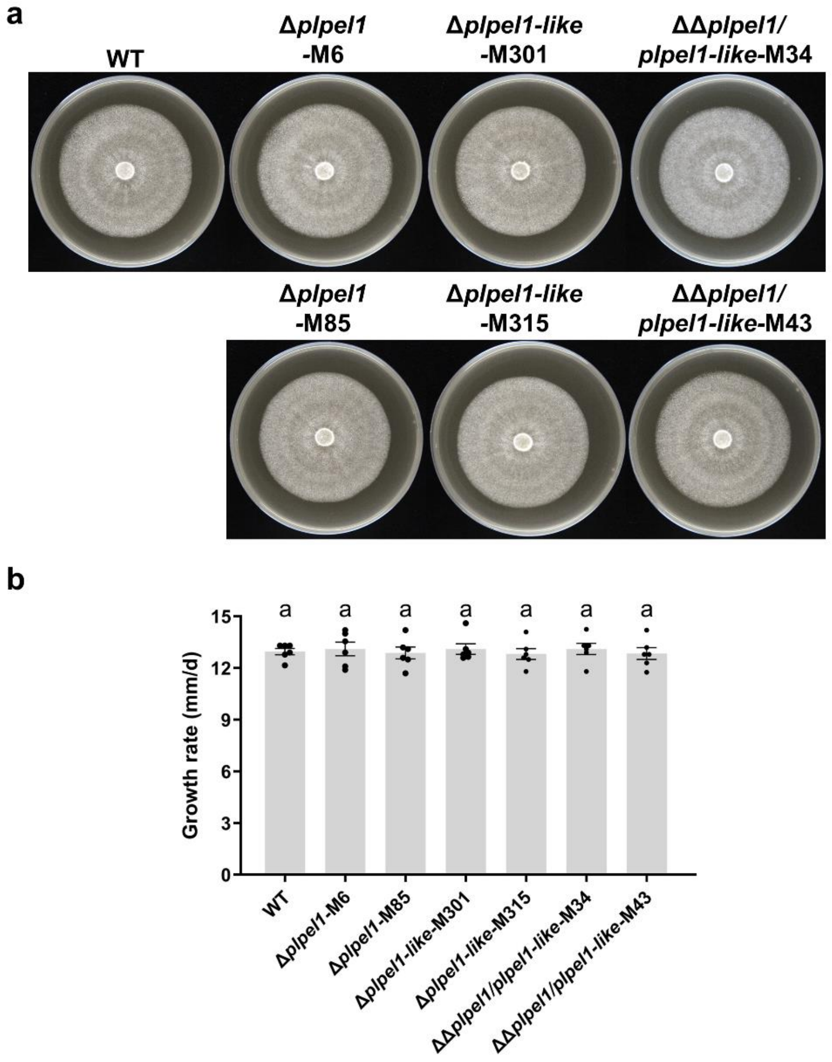

Supplementary Fig. 3. Growth of WT strain and *PlPeL* genes deletion mutants.

(a) Colonies of WT and Δ*plpel1*, Δ*plpel1-like*, and Δ*plpel1/plpel1-like* mutants were cultured on CJA medium at 25°C in the dark for 5 days. Δ*plpel1*, Δ*plpel1-like*, and ΔΔ*plpel1/plpel1-like* presented two independent mutants. (b) Growth rates of WT and Δ*plpel1*, Δ*plpel1-like*, and Δ*plpel1/plpel1-like* mutants were measured on the CJA medium. Data are shown as the mean ± SE (n = 6 biologically independent samples). The data were statistically analyzed with one-way ANOVA. These experiments were repeated three times with similar results. Source data are provided as a Source Data file.

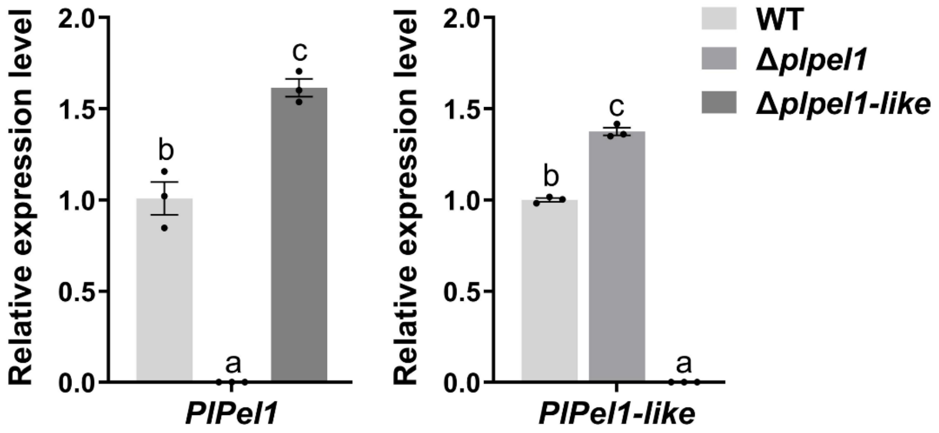

Supplementary Fig. 4. The expression levels of *PlPeL1* and *PlPeL1-like* genes in Δ*plpel1* and Δ*plpel1-like* mutants.

qRT-PCR analyzed the expression levels of *PlPeL1* and *PlPeL1-like* genes in Δ*plpel1*, Δ*plpel1-like* mutants at 12 hpi and their expression levels in WT strain was set as 1. *PlActin* was used as the endogenous control. Data are shown as the mean ± SE of three replicates. Different letters on the graph represent significant differences (One-way ANOVA; $P < 0.05$). These experiments were repeated three times with similar results. Source data are provided as a Source Data file.

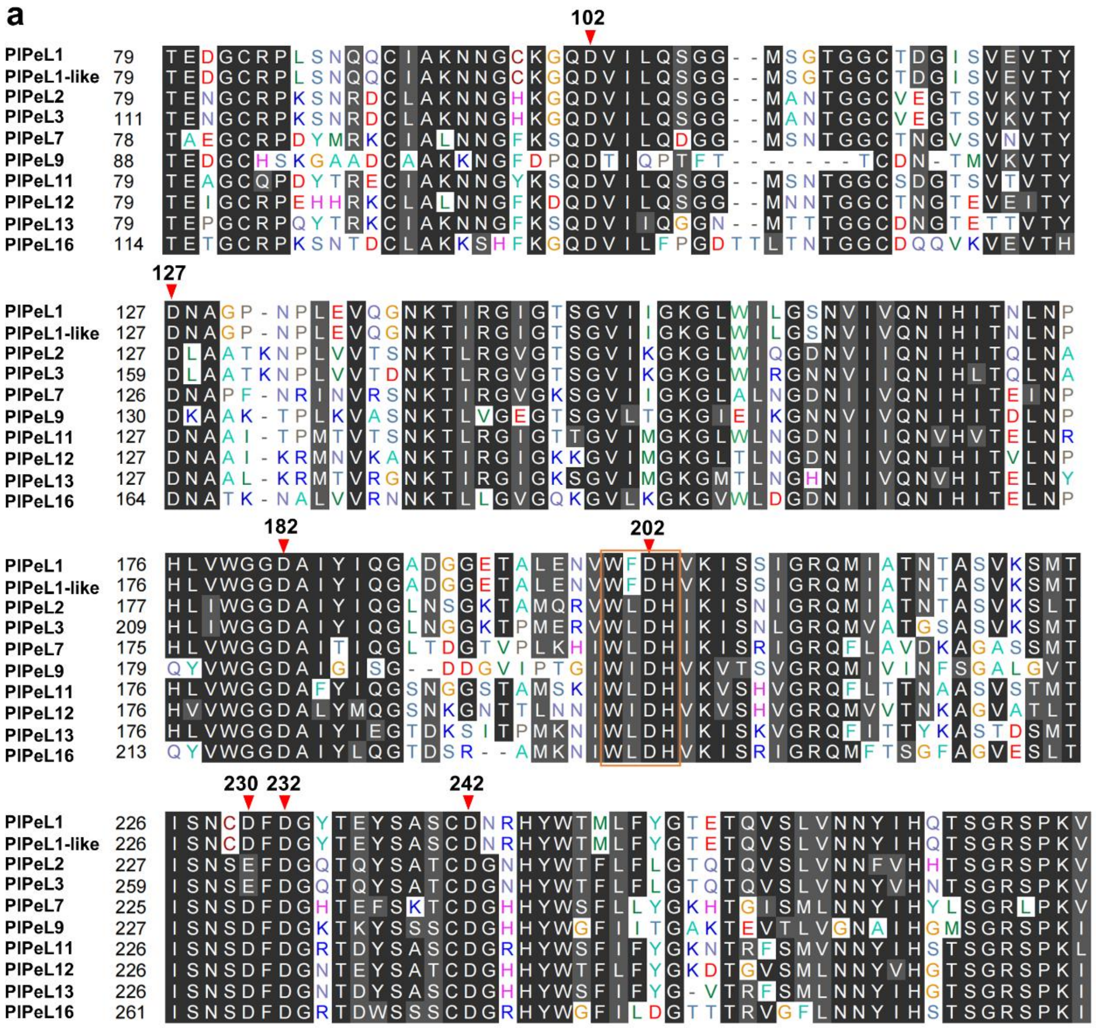
a
102
127
182
202
230 232
242
PlPeL1
PlPeL1-like
PlPeL2
PlPeL3
PlPeL7
PlPeL9
PlPeL11
PlPeL12
PlPeL13
PlPeL16

b

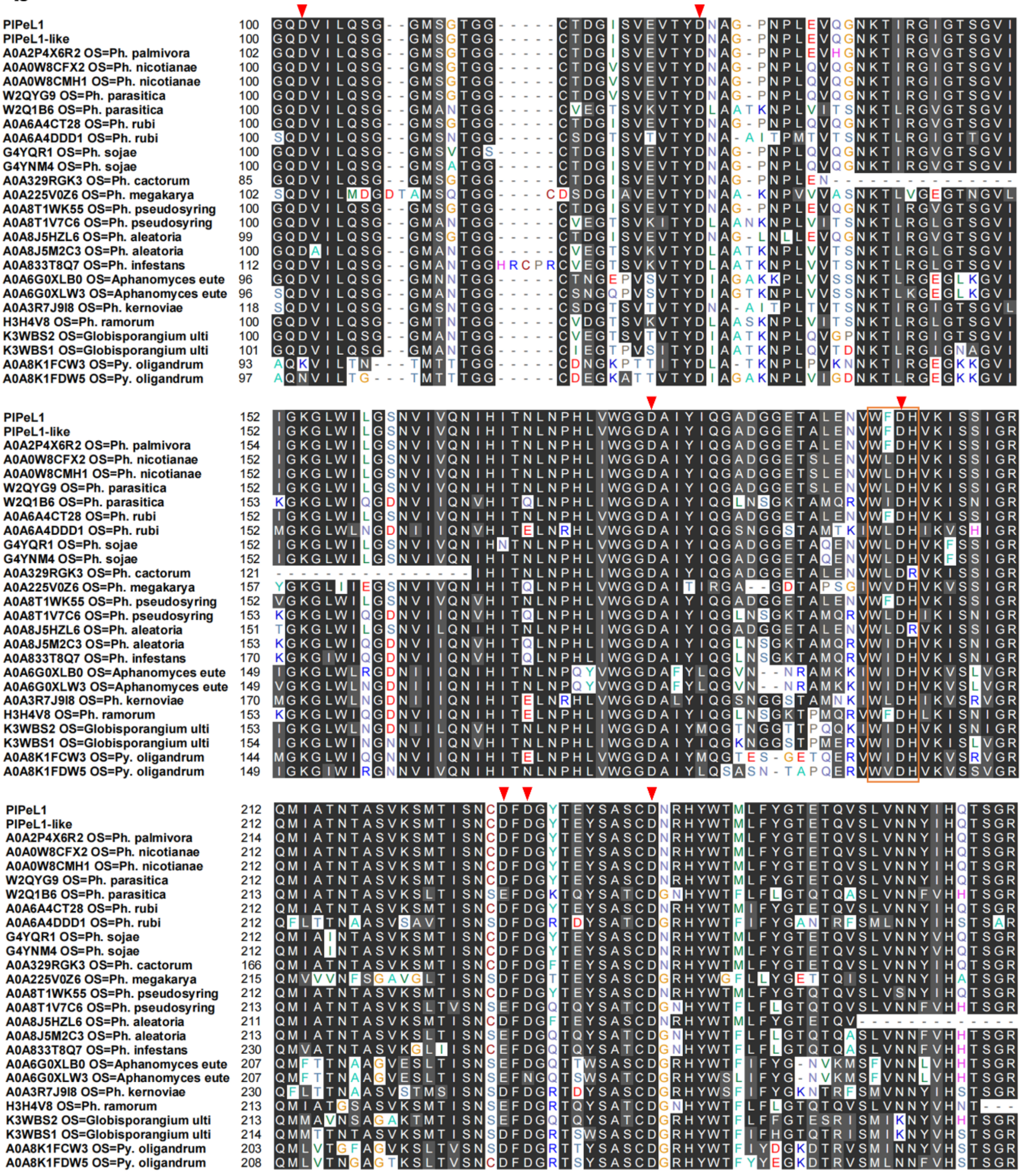

Supplementary Fig. 5. Domain arrangement of 10 PlPeL proteins from *Peronophythora litchii* or 24 homologs of PlPeL1/PlPeL1-like from oomycetes.

(a) Alignment and domain arrangement of 10 PlPeL proteins from *P. litchii*. (b) Alignment and domain arrangement of 24 homologs of PlPeL1/PlPeL1-like from oomycetes. Protein sequence alignments were conducted using ClustalW (BioEdit V7.2.6). Columns containing the same or similar amino acid sequences were colored

black and gray, respectively. The red triangles represent conserved amino acids, aspartic residues, which could be the catalytic center site of Calcium ion (Ca^{2+}). WxDx motif is indicated in orange box.

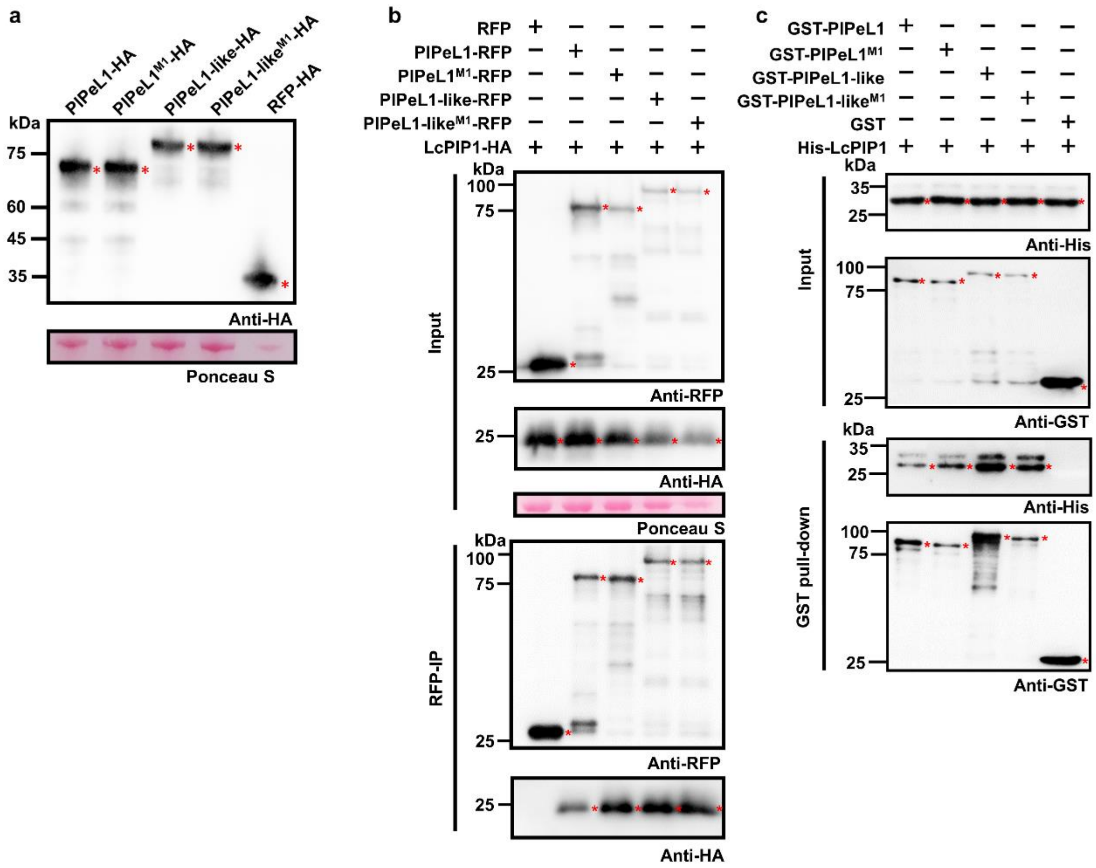

Supplementary Fig. 6. PlPeL1^{M1} and PlPeL1-like^{M1} interact with LcPIP1.

(a) Immunoblot analysis of PlPeL1 and PlPeL1-like mutants expressed in *N. benthamiana* leaves. Total proteins were extracted from *N. benthamiana* leaves at 36 hpa. (b) LcPIP1 interacted with PlPeL1, PlPeL1-like, PlPeL1^{M1}, and PlPeL1-like^{M1} *in planta*. LcPIP1-HA were co-expressed with PlPeL1^{M1}-RFP, PlPeL1-like^{M1}-RFP or RFP in *N. benthamiana* leaves. Protein complexes were immunoprecipitated with RFP-Trap-M beads. Co-precipitation was detected by western blot. (c) LcPIP1 physically interacted with PlPeL1, PlPeL1-like, PlPeL1^{M1}, and PlPeL1-like^{M1} *in vitro*. Co-precipitation was detected by western blot. Red asterisks indicated protein bands of the correct size. Ponceau S staining of Rubisco was used to indicate loading quantity of protein samples. These experiments were repeated three times with similar results.

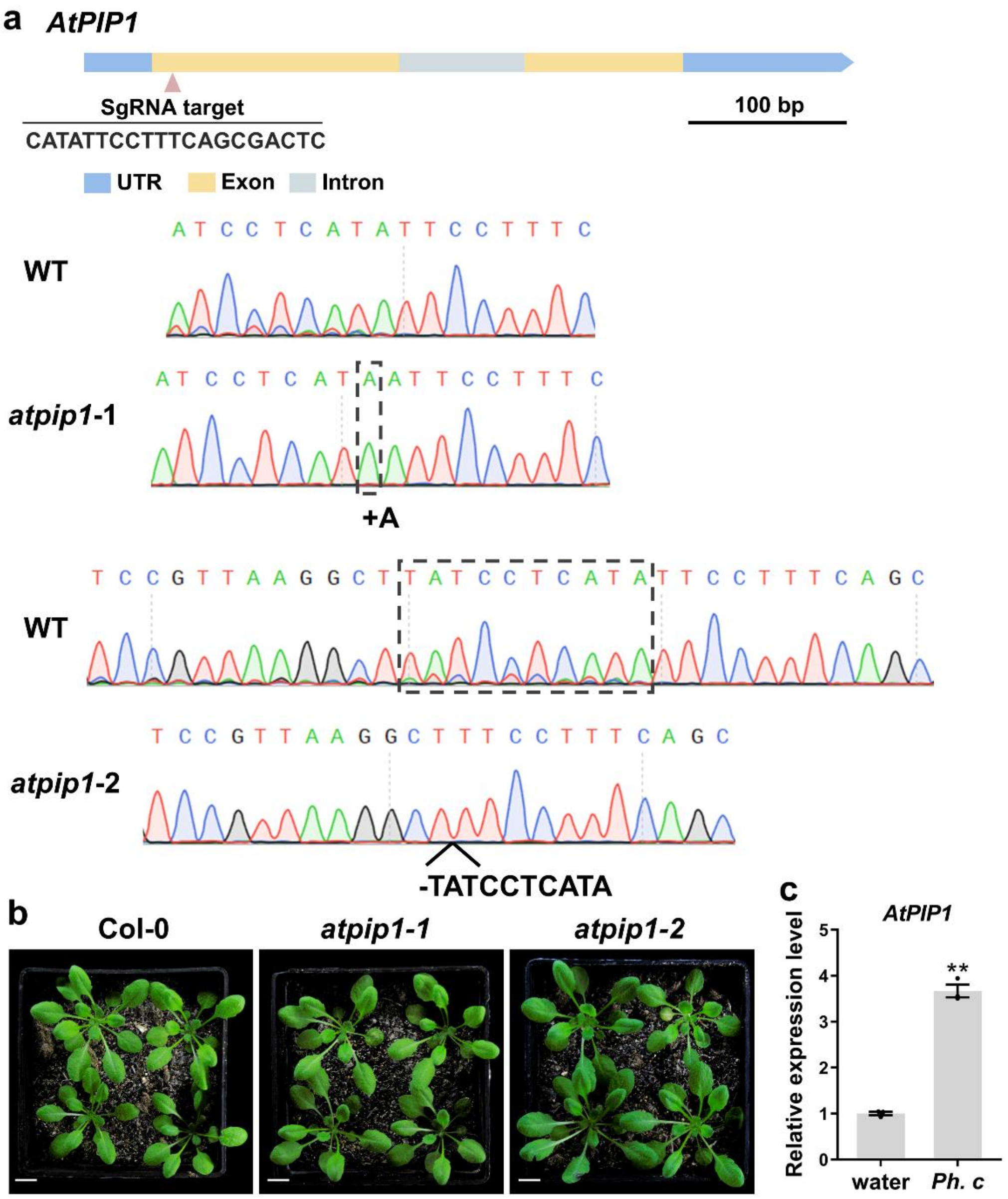

Supplementary Fig. 7. Mutation of *AtPIP1* in *Arabidopsis thaliana* via CRISPR/Cas9-mediated genome editing.

(a) Schematic diagram of single guide RNAs (sgRNA) targeting *AtPIP1*. CRISPR/Cas9-mediated genome editing resulted in the insertion of a single nucleotide at the target site in *atpip1*-1, and a 10-nucleotide deletion at the target site in *atpip1*-2, leading to premature stop codons and amino acid replacements. (b) Growth phenotype of *atpip1* mutants. The growth phenotype of *atpip1* mutants (*atpip1-1* and *atpip1-2*)

and Col-0 were observed at 4 weeks after germination. (c) qRT-PCR analysis of *AtPIP1* expression in response to *Ph. capsici*. Total RNAs were extracted from the *Arabidopsis* leaves inoculated with *Ph. capsici* or water at 36 hpi. The expression level of *AtPIP1* in leaves inoculated with water was set at 1. *AtUBC9* was used as the endogenous control. Data are shown as the mean ± SE of three replicates. Asterisks represent significant difference (Two-tailed Student's *t*-test; $**P < 0.01$). This experiment was repeated three times with similar results. Source data are provided as a Source Data file.

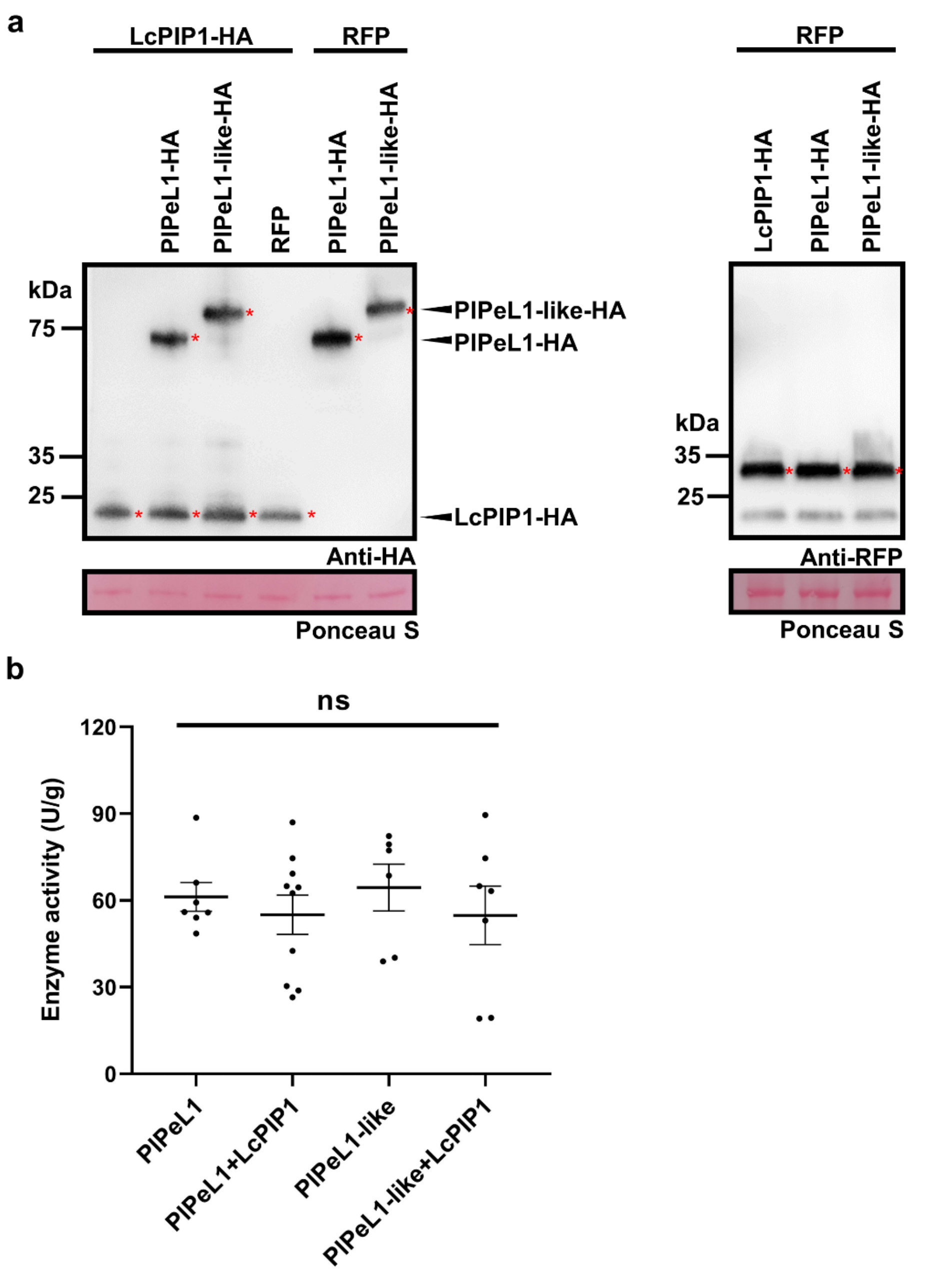

Supplementary Fig. 8. LcPIP1 does not interfere with PlPeL1/PlPeL1-like enzyme activity.

(a) Immunoblot analysis of proteins expressed in *N. benthamiana* leaves. Total proteins were extracted from *N. benthamiana* leaves at 36 hpa. Red asterisks indicated protein bands of the correct size. Ponceau S staining of Rubisco was used to indicate loading quantity of protein samples. (b) PlPeL1 or PlPeL1-like was expressed with LcPIP1 in *N. benthamiana* leaves using agroinfiltration and pectate lyase activity was analyzed at

36 hpa. Data are shown as the mean ± SE (n = 6-10 biologically independent samples). The data were statistically analyzed with one-way ANOVA. These experiments were repeated three times with similar results. Source data are provided as a Source Data file.

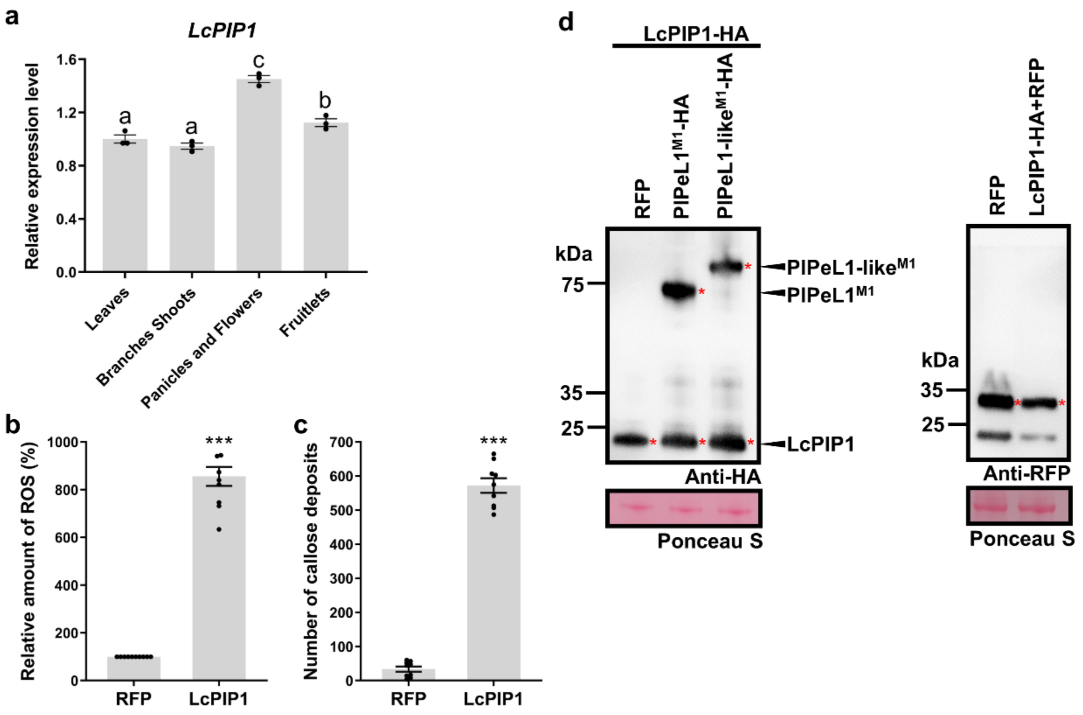

Supplementary Fig. 9. ROS accumulation and callose deposition in *N. benthamiana* leaves expressing *LcPIP1*.

(a) LcPIP1 was ubiquitously expressed in litchi tissues. qRT-PCR analysis of *LcPIP1* expression levels in different organs, including leaves, branches shoots, panicles and flowers, and fruitlets. The relative expression levels were calculated by using the leaves sample as a reference. *LcActin* was used as the endogenous control. Data are shown as the mean ± SE of three replicates. Different letters on the graph represent significant differences among samples (One-way ANOVA; $P < 0.05$). (b,c) Reactive oxygen species (ROS) accumulation and callose deposition observed in LcPIP1-expressing *N. benthamiana* leaves at 2 dpa and RFP expression served as a control. Quantification of the ROS or callose deposits using ImageJ software. Data are shown as the mean ± SE (n = 9-10 biologically independent samples). Asterisks represent significant difference (Two-tailed Student's *t*-test; $^{***}P < 0.001$). (d) Immunoblot analysis of proteins

expressed in *N. benthamiana* leaves. Total proteins were extracted from *N. benthamiana* leaves at 36 hpa. Red asterisks indicated protein bands of the correct size. Ponceau S staining of Rubisco was used to indicate loading quantity of protein samples. These experiments were repeated three times with similar results. Source data are provided as a Source Data file.

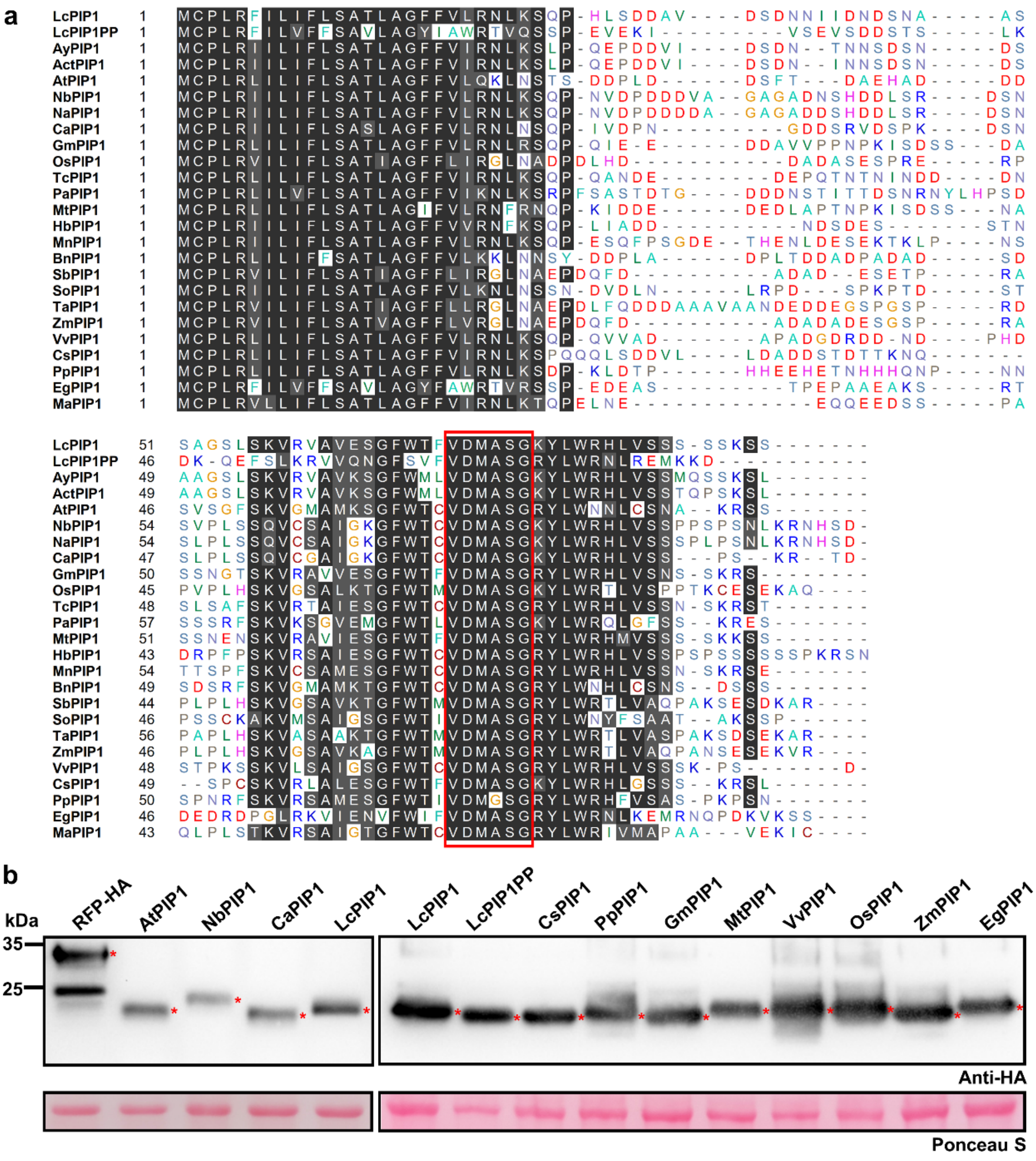

Supplementary Fig. 10. Sequence alignment of LcPIP1 homologs, and western blot confirmed their expression in *Nicotiana benthamiana*.

(a) Sequence alignment of 23 LcPIP1 homologs protein from land plants. LcPIP1 homologs from *Acer yangbiense* (AyPIP1), *Acer truncatum* (ActPIP1), *Arabidopsis thaliana* (AtPIP1), *Nicotiana benthamiana* (NbPIP1), *Nicotiana attenuate* (NaPIP1), *Capsicum annuum* (CaPIP1), *Glycine max* (GmPIP1), *Oryza sativa* (OsPIP1), *Theobroma cacao* (TcPIP1), *Populus alba* (PaPIP1), *Medicago truncatula* (MtPIP1),

Hevea brasiliensis (HbPIP1), *Morus notabilis* (MnPIP1), *Brassica napus* (BnPIP1), *Sorghum bicolor* (SbPIP1), *Spinacia oleracea* (SoPIP1), *Triticum aestivum* (TaPIP1), *Zea mays* (ZmPIP1), *Vitis vinifera* (VvPIP1), *Citrus sinensis* (CsPIP1), *Prunus persica* (PpPIP1), *Eucalyptus grandis* (EgPIP1), and *Musa acuminata* (MaPIP1). LcPIP1PP is a LcPIP1 paralogous protein in litchi. Protein sequence alignments were conducted using ClustalW (BioEdit V7.2.6). The red box indicates the conserved "VDMA/GSG" motif in all LcPIP1 homologs. (b) Western blot confirmed the expression of LcPIP1 homologs in *N. benthamiana* leaves using anti-HA antibody. Red asterisks indicated protein bands of the correct size. Ponceau S staining of Rubisco was used to indicate loading quantity of protein samples. These experiments were repeated three times with similar results.

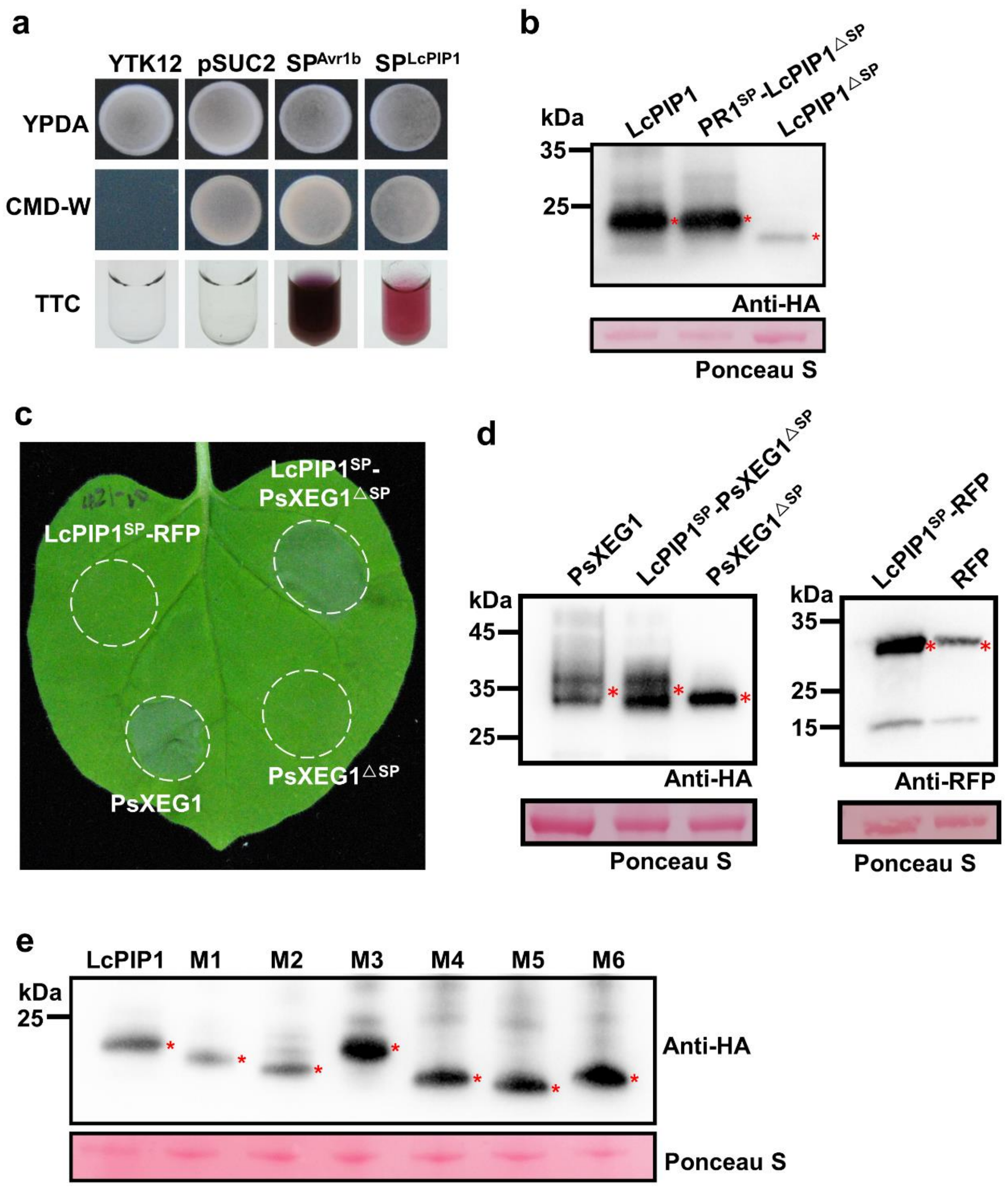

Supplementary Fig. 11. The signal peptide of LcPIP1 possesses secretion function.

(a) Secretion function analysis of LcPIP1 signal peptide (SP). The predicted SP of *LcPIP1* was cloned into the pSUC2 vector. The yeast strain YTK12 carrying the pSUC2 vector can grow on CMD-W medium. SPs of LcPIP1 and Avr1b (positive control) were fused to mature yeast invertase, converting triphenyltetrazolium chloride (TTC) to red 1,3,5-Triphenylformazan. YTK12 and YTK12 carrying the empty as negative controls.

(b) Western blot confirmed the expression of LcPIP1-HA, PR1^{SP}-LcPIP1$^{\triangle SP}$-HA, and LcPIP1$^{\triangle SP}$-HA in *N. benthamiana* leaves using anti-HA antibody. (c) PsXEG1 without SP (PsXEG1$^{\triangle SP}$) failed to trigger cell death, but the SP of LcPIP1 restored the ability of PsXEG1$^{\triangle SP}$ to trigger cell death. Photographs were taken at 4 days post-agroinfiltration (dpa). (d) Western blot confirmed the expression of PsXEG1-HA, LcPIP1^{SP}-PsXEG1$^{\triangle SP}$-HA, and PsXEG1$^{\triangle SP}$-HA in *N. benthamiana* leaves using anti-HA antibody. The expression of LcPIP1^{SP}-RFP and RFP in *N. benthamiana* leaves using anti-RFP antibody. (e) Western blot confirmed the expression of LcPIP1 mutants (M1-M6) using anti-HA antibody. Red asterisks indicated protein bands of the correct size. Ponceau S staining of Rubisco was used to indicate loading quantity of protein samples. These experiments were repeated three times with similar results.

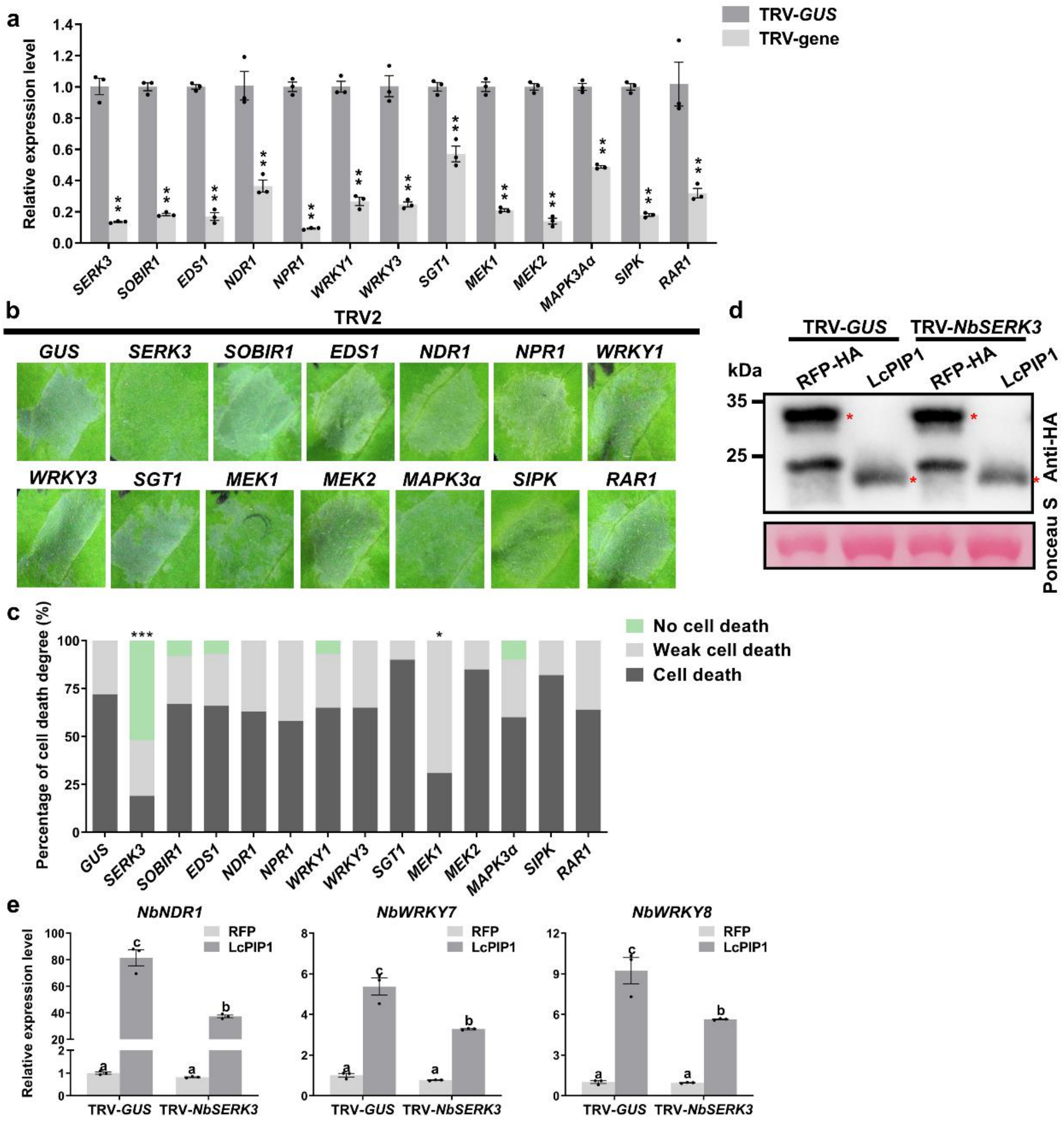

Supplementary Fig. 12. Analysis of the plant immunity components involved in LcPIP1-induced cell death.

(a) The transcript abundance of *SERK3*, *SOBIR1*, *EDS1*, *NDR1*, *NPR1*, *WRKY1*, *WRKY3*, *SGT1*, *MEK1*, *MEK2*, *MAPK3α*, *SIPK*, and *RAR1* in corresponding silenced plants was determined by qRT-PCR. The *NbEF1α* was used as the endogenous control. Data are shown as the mean ± SE of three replicates. Asterisks represent significant difference (Two-tailed Student's *t*-test; $**P < 0.01$). (b) LcPIP1 was expressed in these silenced plants and *GUS*-silenced plants were used as negative controls. Photographs

were taken at 4 dpa. (c) Quantification of cell death in corresponding plants. The degree of cell death was divided into three levels: no cell death, weak cell death, and strong cell death. Asterisks represent significant differences (***P<0.001, *P < 0.05) based on Wilcoxon rank-sum test. (d) Protein expression was verified by western blot. Red asterisks indicated protein bands of the correct size. Ponceau S staining of Rubisco was used to indicate loading quantity of protein samples. (e) qRT-PCR was used to analyze the expression levels of *NbNDR1*, *NbWRKY7*, and *NbWRKY8* in *NbSERK3*- or *GUS*-silenced plants expressing LcPIP1 or RFP at 48 hpa. *NbEF1α* was used as the endogenous control. Data are shown as the mean ± SE of three replicates. Different letters on the graph represent significant differences among samples (One-way ANOVA; P < 0.05). These experiments were repeated three times with similar results. Source data are provided as a Source Data file.

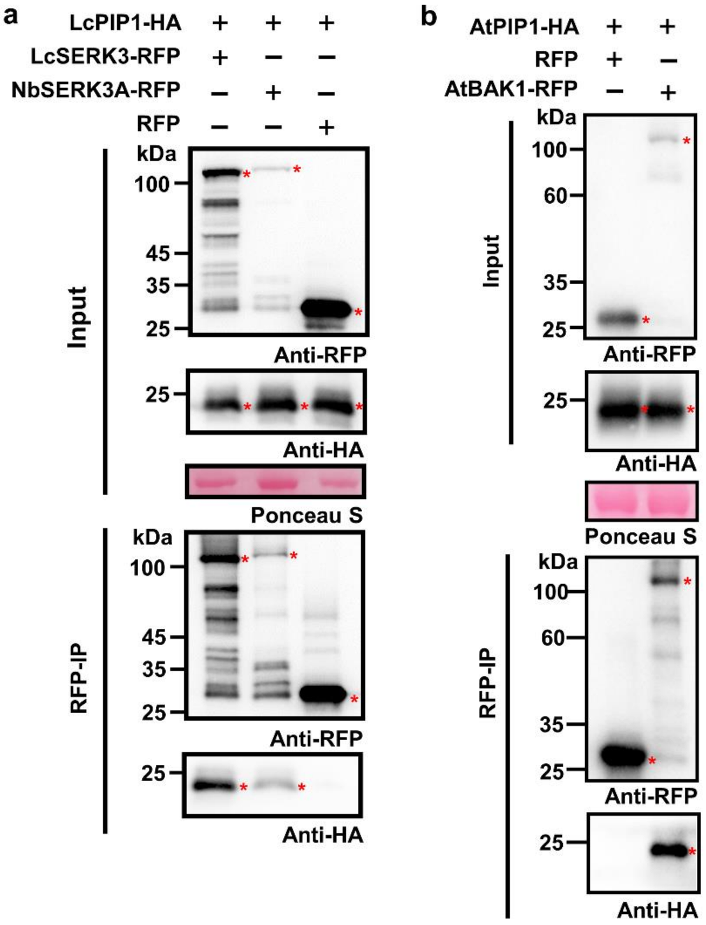

Supplementary Fig. 13. SERK3 (BAK1) interact with PIP1s.

(a) LcPIP1-HA was co-expressed with LcSERK3-RFP, NbSERK3A-RFP, or RFP in *N. benthamiana* leaves. Protein complexes were immunoprecipitated with RFP-Trap-M beads. Co-precipitation was detected by western blot. (b) AtPIP1 interacted with AtBAK1 *in planta*. AtPIP1-HA was co-expressed with AtBAK1-RFP or RFP in *N. benthamiana* leaves. Protein complexes were immunoprecipitated with RFP-Trap-M beads and detected by western blot. Red asterisks indicated protein bands of the correct size. Ponceau S staining of Rubisco was used to indicate loading quantity of protein samples. These experiments were repeated three times with similar results.

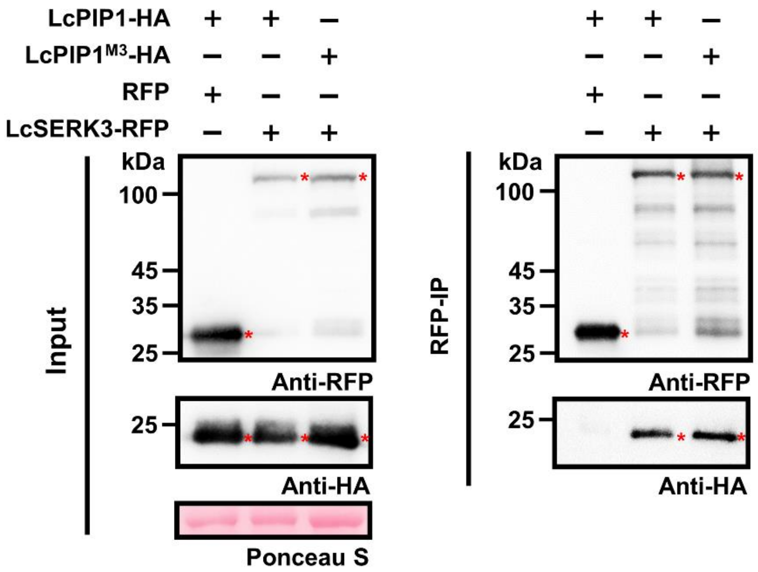

Supplementary Fig. 14. LcSERK3 interacts with LcPIP1 and LcPIP1^{M3} *in planta*. LcPIP1-HA or LcPIP1^{M3}-HA were co-expressed with LcSERK3-RFP or RFP in *N. benthamiana* leaves. Protein complexes were immunoprecipitated with RFP-Trap-M beads. Co-precipitation was detected by western blot. Red asterisks indicated protein bands of the correct size. Ponceau S staining of Rubisco was used to indicate loading quantity of protein samples. These experiments were repeated three times with similar results.

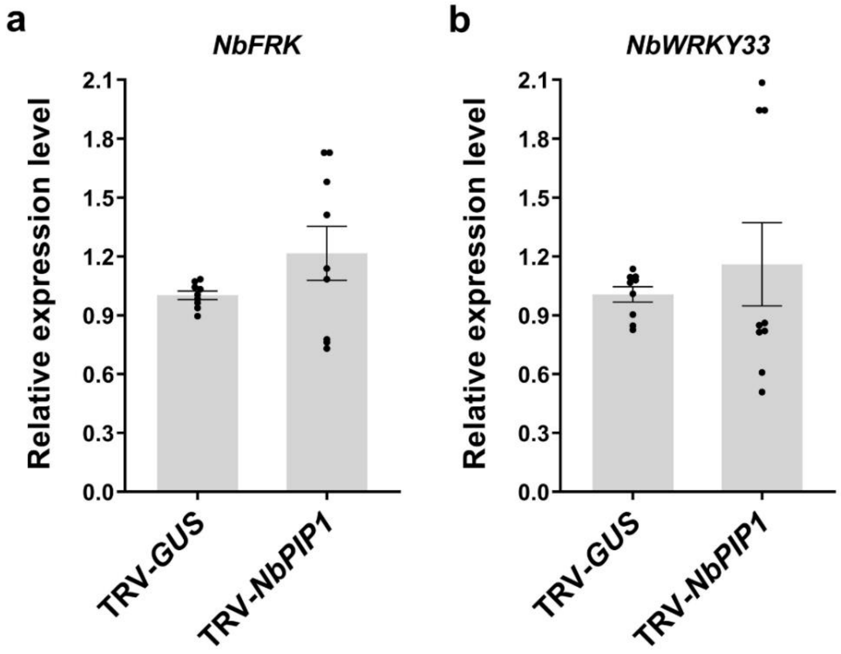

Supplementary Fig. 15. NbPIP1 does not regulate flg22-triggered PTI responses. qRT-PCR was used to analyze the expression levels of *NbFRK* and *NbWRKY33* in *NbPIP1- or GUS-* silenced plants after treatment with 10 μM flg22 for 3 h. The expression level of *NbFRK* or *NbWRKY33* in *GUS*-silenced plants treated with flg22 was set at 1. The *NbEF1α* was used the endogenous control. Data are shown as the mean ± SE of nine replicates. Two-tailed Student's *t*-test was used for significance analysis. These experiments were repeated three times with similar results. Source data are provided as a Source Data file.
