# Supplementary material for: A plant cell death-inducing protein from litchi interacts with Peronophythora litchii pectate lyase and enhances plant resistance
Source: Nat Commun. 2024 Jan 2;15:22. doi: 10.1038/s41467-023-44356-y (PMC10761943; doi:10.1038/s41467-023-44356-y)
Supplement: Supplementary file 3 — Description of Additional Supplementary Files [file 41467_2023_44356_MOESM3_ESM.pdf]

Description of Additional Supplementary Files:

Supplementary Data 1: Ten PlPeL proteins form Peronophythora litchii SHS3.

Supplementary Data 2: Homologous proteins of PlPeL1/PlPeL1-like in oomycete.

Supplementary Data 3: Homologous proteins of LcPIP1 in plants.

Supplementary Data 4: The primers used in this study.

Supplementary Data 5: Plasmid constructs used in this study.
